# Human Identification by Cross-Correlation and Pattern Matching of Personalized Heartbeat: Influence of ECG Leads and Reference Database Size

**DOI:** 10.3390/s18020372

**Published:** 2018-01-27

**Authors:** Irena Jekova, Vessela Krasteva, Ramun Schmid

**Affiliations:** 1 Institute of Biophysics and Biomedical Engineering, Bulgarian Academy of Sciences, Acad. G. Bonchev Str. Bl 105, 1113 Sofia, Bulgaria; vessika@biomed.bas.bg; 2 Schiller AG, Signal Processing, 6341 Baar, Switzerland; ramun.schmid@schiller.ch

**Keywords:** electrocardiogram, ECG based biometrics, person identification, correlation, template matching, PQRST pattern, QRS pattern

## Abstract

Human identification (ID) is a biometric task, comparing single input sample to many stored templates to identify an individual in a reference database. This paper aims to present the perspectives of personalized heartbeat pattern for reliable ECG-based identification. The investigations are using a database with 460 pairs of 12-lead resting electrocardiograms (ECG) with 10-s durations recorded at time-instants T1 and T2 > T1 + 1 year. Intra-subject long-term ECG stability and inter-subject variability of personalized PQRST (500 ms) and QRS (100 ms) patterns is quantified via cross-correlation, amplitude ratio and pattern matching between T1 and T2 using 7 features × 12-leads. Single and multi-lead ID models are trained on the first 230 ECG pairs. Their validation on 10, 20, ... 230 reference subjects (RS) from the remaining 230 ECG pairs shows: (i) two best single-lead ID models using lead II for a small population RS = (10–140) with identification accuracy *AccID* = (89.4–67.2)% and aVF for a large population RS = (140–230) with *AccID* = (67.2–63.9)%; (ii) better performance of the 6-lead limb vs. the 6-lead chest ID model—(91.4–76.1)% vs. (90.9–70)% for RS = (10–230); (iii) best performance of the 12-lead ID model—(98.4–87.4)% for RS = (10–230). The tolerable reference database size, keeping *AccID* > 80%, is RS = 30 in the single-lead ID scenario (II); RS = 50 (6 chest leads); RS = 100 (6 limb leads), RS > 230—maximal population in this study (12-lead ECG).

## 1. Introduction

Biometric identity recognition systems using human body measurement data are vital for the security of financial transactions, access control, travel, etc. The various biometrics that are currently being adopted in identity recognition scenarios such as fingerprint, iris, face and speech recognition have operational trade-offs in terms of performance, measurability, circumvention and liveness detection [1]. These limitations have led to the development of smart solutions such as: (i) improving voice recognition by sophisticated pre-processing, followed by heuristical selection of significant features in time and frequency domains [2]; (ii) integrating face- and body-related soft biometric traits [3]; (iii) providing security and flexibility by simultaneous application of numeric password data and biometric fingerprint information [4]; (iv) assuring liveness detection via optical sensors, which establish the presence of pulse, the variations of optical characteristics caused by pressure changes and skin reaction to illumination with different wavelengths [5]. 

For more than a decade the electrocardiogram (ECG) has been intensively investigated in the context of biometric applications. The attention is attracted by its ability to provide an unambiguous sign of liveness and to exhibit several personalized traits, reflecting the underlying physiological properties of the heart such as relative timings of the P, QRS, T waves and differences in beat geometry. An additional precondition for the use of biomedical data for identification purposes is the rapid development of multidimensional sensors making it possible to efficiently acquire such data [6,7]. Recently, the strategic application of biometric systems has been extended towards:

- remote healthcare monitoring [8,9] with biosensors integrated into mobile devices [10];
- secure wireless body area sensor networks [11,12];
- continuous authentication applications with adaptive strategies to follow individual beat variations in 24 h ECG recordings [13];
- continuous human recognition in driving settings [14].

Similar to any other biometric system, the operation of those, embedding ECG analysis, could be spitted into two phases [15]:

- Enrolment or registration phase, when a reference database from subjects with known identity is collected;
- Matching phase with two possible scenarios:
  - Person verification, when the system accepts or rejects someone’s identity based on a single comparison to the data for a given ID in the reference database (one-to-one scenario);
  - Person identification, when the system is finding the identity of an individual based on a search in a reference database for the subject whose data best matches the input data (one-to-many scenario).

In terms of feature extraction methods, identification techniques based on ECG analysis could be assigned into three categories:

- Fiducial based approaches, which analyse:
  - Temporal, amplitude, area and angle features over single lead [9] or 12-lead ECG [16];
  - Only temporal features measured in a single lead that are hypothesized to be invariant to sensor placement [12,17,18].

The accuracy of these methods strongly depends on the correct localization of the waves’ boundaries within the P-QRS-T segment.

- Non-fiducial methods based on calculation of:
  - Autocorrelation functions and their post-processing via discrete cosine transform [14,19];
  - Linear discriminant analysis [20] for feature space reduction and further identification;
  - Assessment of correlation coefficients between beats with known and unknown identity [21,22,23];
  - Pattern matching and estimation of normalized Euclidian distance between the tested and the target heartbeat [24];
  - Features describing the dynamic characteristics and chaotic behaviour in the ECG time series [25].
- Hybrid human identification methods combining both fiducial and non-fiducial strategies, which include:
  - Hierarchical identification scheme for temporal and amplitude measurements based on fiducial points detection and estimation of waveforms‘ similarity via Euclidean distance [26];
  - Fiducial-based measurements for reduction of the matching candidates’ number followed by wavelet transform and application of the coefficients for template matching [8].

Numerous studies are focused on general-purpose practical applications limited to the use of a single ECG lead, usually from the limbs [10,17,18,23,24]. Others aim at effective channel combination schemes by involving 12-lead ECG [16,20,21,22] into the analysis in view of feasible applications in healthcare scenarios. The merit of multi-lead ECG is controversial, considering: on the one hand the identification accuracy improvement by increasing the analysed ECG leads in a fiducial independent application [22] and on the other hand the same level of identification accuracy for all 12 leads, only limb, only chest and only lead I in a fiducial-based application [16]. 

Despite that the majority of the studies have used public [9,10,19,20,23,26] or proprietary [10,16,17,18,21,22,23,24] clinical databases, some authors have addressed the application of different non-invasive portable single-channel ECG sensors which support the acquisition of ECG signals for human identification purposes. The reported identification accuracies are:

- ~99% for 30 ECG signals acquired from the individual via a biosensor integrated into mobile devices [10];
- ~90% for 30 ECG test patterns acquired from the palms of 9 subjects via a two poles self-developed ECG signal acquisition system [25];
- ~94% achieved for ECG recordings acquired from the fingers of 16 individuals via self-developed ECG sensor [27].

The use of such ECG sensors facilitates the practical application of ECG biometrics in areas such as cyber-security [25,27], cloud data security or remote healthcare systems [10].

The majority of the cited methods report high identification accuracy. However, certain limitations are observed such as:

- Small population size (<30 individuals) [16,17,18,19,20,21,22,25,26,27] with ECG recordings acquired in a very short temporal interval [16,21,22] or even during the same session [9,17,18,20,26];
- Application of one and the same database for both training and testing.

This is far from the realm of biometrics. Significant accuracy degradation could be expected [28] with these methods, due to the influence of different unpredictable factors such as long-term ECG changes [13], noisy environment [14], electrode misplacement, overtraining of the identification model, etc. 

The aim of this paper is to present the 12-lead ECG as a biometric modality by describing three methods for comparison between input-to-template waveforms of personalized beat patterns based on cross-correlation, amplitude relations and a new method for binary pattern matching. We further investigate their potential for human identification within an uncommonly large population with one-year distant collection of the reference and test datasets. Unbiased statistical analysis on independent validation set is presented for different perspectives of the human identification problem, considering: (i) the choice of the optimal single and multi-lead ECG set; (ii) the influence of the reference database size, providing evidence about the reasonable size of the population that could be tolerated in ECG biometric studies.

## 2. Materials and Methods

### 2.1. ECG Database

The study is using a proprietary clinical ECG database (Schiller AG, Baar, Switzerland) provided for research purposes and for the investigation of ECG’s potential for human biometrics on a large population observed over time. It contains two 10-s sessions of standard 12-lead resting ECGs from 460 non-cardiac patients (235/225 male/female, 18–106 years old) admitted in the emergency department of the Basel University Hospital between 2004 and 2009. The ECGs were recorded via a commercial ECG device (SCHILLER AT-110 (500 Hz, 2.5 µV/LSB, 0.05 Hz–150 Hz bandwidth), Schiller AG, Baar, Switzerland) at different time-points, i.e. reference point T1, corresponding to the enrolment phase of the authentication task and remote point T2 > T1 + 1 year, corresponding to the matching/login phase. The 460 ECG couples were equally divided into two datasets from non-overlapping populations for independent training and validation of the designed human identification models:

- Training dataset, including the ECG couples from 1 to 230;
- Validation dataset, including the ECG couples from 231 to 460.

### 2.2. Methods

**Figure 1:**
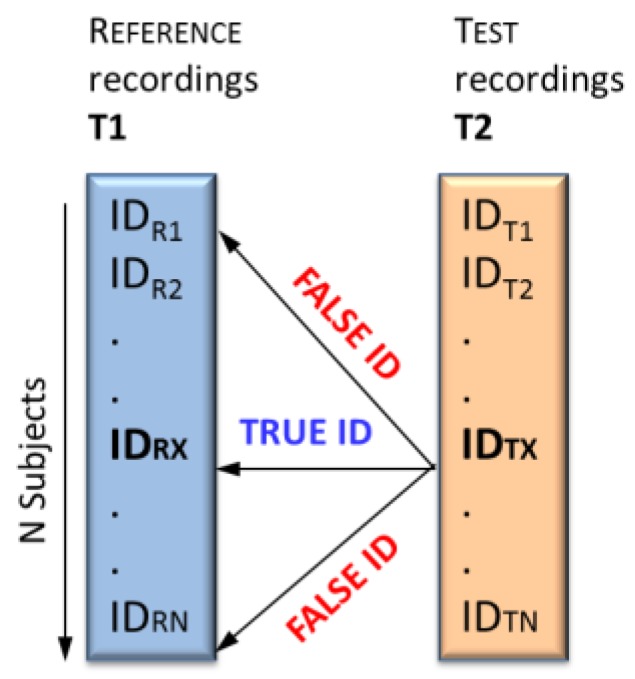
Identification concept scheme illustrating the comparison of the tested recording (ID_TX_) in T2 to all reference recordings in T1 (from ID_R1_ to ID_RN_).

The identification concept (Figure 1) is based on a comparison between a tested subject with an unknown identity (ID_T_) from dataset (T2) and a set of N subjects from a previously recorded reference dataset (T1) with known identities (ID_R_). The purpose in the identification task is to answer the question: “*Who is this person?*”

The identification accuracy (*AccID*) is calculated as follows:

(1) $$AccID=100.\frac{TrueID}{NSubjects}(\%)$$

where the true identifications (*TrueID*) consider the number of all correctly decided cases for which the identity of ID_T_(T2) exactly corresponds to the identity of ID_R_(T1) and *NSubjects* is the number of tested subjects in the reference database.

**Figure 2:**
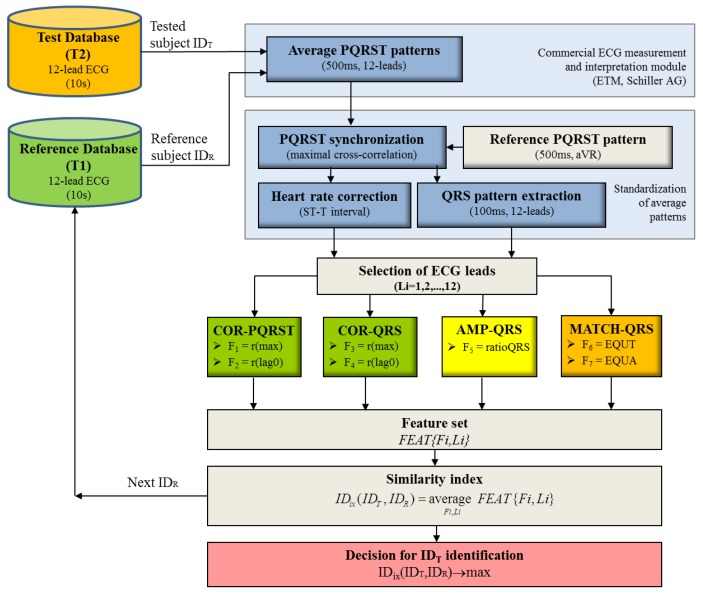
Block-diagram of the identification process representing ECG pre-processing for the extraction of personalized 12-lead average PQRST pattern, features calculation and decision making.

Тhe block-diagram of the module designed for human identification is presented in Figure 2. The ECG pre-processing and the methods for cross-correlation and amplitude features extraction are inherited from our recent study on ECG-based human verification [29]. The novelty in this identification model is generally in the designed brand-new method for QRS pattern matching and the decision making, based on features’ common similarity index. We further describe the methods in detail to facilitate their reproduction.

#### 2.2.1. ECG pre-processing

All ECG recordings are processed by a certified commercial ECG measurement and interpretation module (ETM, Schiller AG, Baar, Switzerland) for the extraction of a 12-lead average PQRST pattern with duration of 500 ms. This pattern, further referred as personalized pattern, provides higher signal-to-noise ratio and greater robustness against respiration-induced morphology changes in ECG as compared to single heartbeats. 

Inter-subject standardization of 12-lead PQRST patterns is applied by:

- Detrending (removal of DC offset and linear trend);
- Synchronization of the cardiac depolarization process by time-alignment of the personalized PQRST pattern to a reference pattern by maximal cross-correlation in lead aVR. The reference pattern was selected at the beginning of the identification study. It represents PQRST segment in lead aVR with normal morphology (negative P, R, T waves) belonging to a healthy subject from the reference database (T1).
- Extraction of QRS pattern (100 ms), synchronously in all 12 leads within a window of 30 ms before and 70 ms after the fiducial point, aligned to the R-peak of the reference pattern. The additional investigations of this short pattern are motivated by the findings in our previous study [30] which highlight the biometric potential of the R and S-waves and reject the verification capability of the P, ST, T parts due to their low intra-subject reproducibility and low inter-subject variability.
- Calculation of heart rate corrected ST-T interval by means of Bazett’s formula $QTc=QT/\sqrt{RR}$, where *QTc* is the corrected *QT* interval and *RR* is the RR interval.

#### 2.2.2. Features extraction

Comparison between the waveforms of the personalized PQRST and QRS patterns of a tested vs. reference subject (ID_T_ vs. ID_R_) is performed independently for each of the 12 leads by three feature extraction methods—cross-correlation analysis of QRS/PQRST and amplitude measurements over the QRS (applied also in [29]) and a brand-new method for assessment of QRS pattern matching between ID_T_ and ID_R_.

(1) Cross-correlation analysis of QRS and PQRST patterns (COR-QRS and COR-PQRST) based on the cross-correlation function *r*:

(2) $$r_{ID_{T},ID_{R}}(lag)=\frac{\sum_{i=1}^{PD}Pattern_{ID_{T}}(i)Pattern_{ID_{R}}(i+lag)}{\sqrt{\sum_{i=1}^{PD}Pattern_{ID_{T}}{\left(i\right)}^{2}\sum_{i=1}^{PD}Pattern_{ID_{R}}{\left(i\right)}^{2}}}$$

where *Pattern* denotes QRS or PQRST with pattern duration *PD* = 100 ms or 500 ms, respectively. The *lag* value is changed in the range [−PD; PD].

Two correlation coefficients are calculated in the normalized scale [0; 100]%: 

*Maximum correlation r*(max) is representative for the best matching of the waveforms:

(3) $$r(\max)=\underset{lag\in [-PD,PD]}{m\mathrm{ax}\;}r_{ID_{T},ID_{R}}(lag)*100(\%)$$

*Zero-lag correlation r*(*lag*0) is representative for the non-synchronized similarity between the ECG patterns:

(4) $$r(lag0)=r_{ID_{T},ID_{R}}(lag=0)*100(\%)$$

Reduced values of both correlation coefficients are expected for subjects with different ID (ID_T_ ≠ ID_R_) due to the different spatio-temporal dynamics of the cardiac vector between subjects.

(2) Amplitude measurements of the QRS patterns (AMP-QRS) estimating the feature:

*Ratio of the minimal-to-maximal QRS amplitude* representative of the peak-to-peak amplitude equality between the QRS patterns in the studied lead, comparing ID_T_ and ID_R_ recordings. 

(5) $$ratioQRS=\frac{\underset{ID=ID_{T},ID_{R}}{\min}QRS_{ID}}{\underset{ID=ID_{T},ID_{R}}{\max}QRS_{ID}}*100(\%)$$

A ratio close to 100% is expected for equal identity subjects ID_T(T1)_ = ID_R(T2)_ considering the same recording conditions in T1 and T2 sessions (e.g. posture, electrode placement, recording ECG device, etc.) and therefore similar QRS amplitudes. 

(3) QRS pattern matching (MATCH-QRS) applying amplitude normalization; time-amplitude approximation; and extraction of features for assessment of QRS pattern matching between ID_T_ and ID_R_. 

Amplitude normalization

The amplitudes of the QRS patterns in any pair (ID_T_, ID_R_.) and any lead are linearly scaled to fit in the range [−1; 1] in order to use one and the same computational range for all individuals and all leads regardless of their signal amplitudes. This is achieved by dividing each sample of the QRS patterns in a particular ECG lead to the maximal absolute amplitude observed in this lead among *QRS_IDT_* and *QRS_IDR_*. 

Time-amplitude approximation

The QRS waveforms of ID_T_ and ID_R_ are approximated by the following binary transform (*binQRS_ID_*), which converts the QRS pattern into a 2-dimensional matrix:

(6) $$binQRS_{ID}(ti,aj)=\{\begin{array}{ll} 1 & \mathrm{if}\;A(aj)\in [\;QRS_{ID}(ti)\pm 2\Delta a]\;\mathrm{or}\;A(aj)\in [\;QRS_{ID}(ti\pm \Delta t)]\;\\ 0 & \mathrm{otherwise} \end{array}$$

where

- *ti* = [1, 2, ... 100] is the index of the time scale *T*(*ti*) = [1, 1 + Δ*t*, 1 + 2Δ*t*, …, 100] ms with resolution Δ*t* = 1 ms.
- *aj* = [1, 2, … 80] is the index of the normalized amplitude scale *A*(*aj*) = [−1, −1+ Δ*a*, −1 + 2Δ*a*, …, 1] with resolution Δ*a* = 0.025.

The introduced tolerances *ti* ± 1 ms and *A*(*aj*) ± 0.05 manage small intra- and inter-subject variations of the cardiac depolarization process. Larger values of Δ*t* and Δ*a* would lead to a rougher approximation of the QRS patterns and potential loss of waveform details while smaller values support finer approximation at the cost of increased computational resources.

Extraction of pattern matching features

Two MATCH-QRS features are calculated for the couple (ID_T_, ID_R_), normalized in the scale [0; 100]%:

*Measure of the equality in the time scale (EQUT)* representing the time of overlapping of both QRS patterns after binary element-wise multiplication (AND operation):

(7) $$EQUT=\frac{\Delta t}{100ms}\sum_{ti=1}^{100}binQRS_{ID_{T}}^{}(ti,aj)\wedge binQRS_{ID_{R}}^{}(ti,aj)*100(\%)\;\mathrm{for}\;aj=(1—80)$$

*EQUT* ranges between 0% (null time coincidence, i.e. patterns do not overlap for any *binQRS* entry over the complete pattern length) and 100% (full-time coincidence, i.e. patterns overlap for all entries over the complete pattern length). The largest *EQUT* values are expected for equal identity subjects ID_T_ = ID_R_.

*Measurement of the equality in the amplitude scale (EQUA):*

(8) EQUA=100−DIFA(%)

where *DIFA* (difference in the amplitude scale) represents the area enclosed between the non-overlapping amplitudes of both QRS patterns after binary element-wise multiplication and inversion (NAND operation):

(9) $$DIFA=\frac{\Delta t}{100ms}\Delta a\sum_{ti=1}^{100}\sum_{aj=a\min}^{a\max}binQRS_{ID_{T}}(ti,aj)\bar{\wedge }binQRS_{ID_{R}}^{}(ti,aj)*100(\%)$$

The integration interval in the amplitude scale is enclosed between the minimal and maximal QRS amplitudes among ID_T_ and ID_R_ patterns measured at each specific time index *ti*, i.e., $\left[a\min(ti)=\underset{ID=ID_{R},ID_{T}}{\min\;}binQRS_{ID}(ti\right)$; $\left[a\max(ti)=\underset{ID=ID_{R},ID_{T}}{\max\;}binQRS_{ID}(ti\right)$]. *DIFA* ranges between 0% (full-amplitude coincidence, i.e., patterns overlap for all binQRS entries over the complete pattern length) and 100% (null amplitude coincidence, corresponding to the largest pattern differences that cover the full amplitude range). Therefore, minimal *DIFA* and inversely maximal *EQUA* values are expected for equal identity subjects ID_T_ = ID_R_.

The redundant input feature space is a matrix *FEAT*{*Fi*, *Li*}, where:

- *Fi* = (1–7) is the index in the feature vector {*r*(max)*_PQRST_*, *r*(*lag*0)*_PQRST_*, *r*(max)*_QRS_, r*(*lag*0)*_QRS_*, *ratioQRS*, *EQUT*, *EQUA*}, including the option to enable/disable any feature from analysis and thus to test ECG identification scenarios with different feature sets.
- *Li* = (1–12) is the index of the lead including the option to enable/disable any lead from analysis and thus to test different single- and multi-lead configurations for ECG identification.

#### 2.2.3. Decision Making

For the purpose of human identification, the best matching between ID_T_ and ID_R_ is found by maximization of the similarity index:

(10) $$ID_{ix}=\underset{Fi,Li}{\mathrm{average}\;}FEAT\{Fi,Li\}$$

Optimal identification (ID) models with non-redundant features are trained by forward stepwise feature selection until maximization of accuracy *AccID* on the training dataset. The observed accuracy on the independent validation set could be considered as unbiased assessment of the human identification models.

The software package Matlab (The Mathworks Inc., Natick, MA, USA) was used for the management of the signal processing and statistical study. This includes training and validation of the forward stepwise ID models (reported as mean ± confidence interval (CI)) as well as for comparison of continuous feature distributions (represented as mean ± standard deviation (std)) via paired Student’s *T*-Test. A value of *p* ≤ 0.05 is considered statistically significant.

## 3. Results

**Figure 3:**
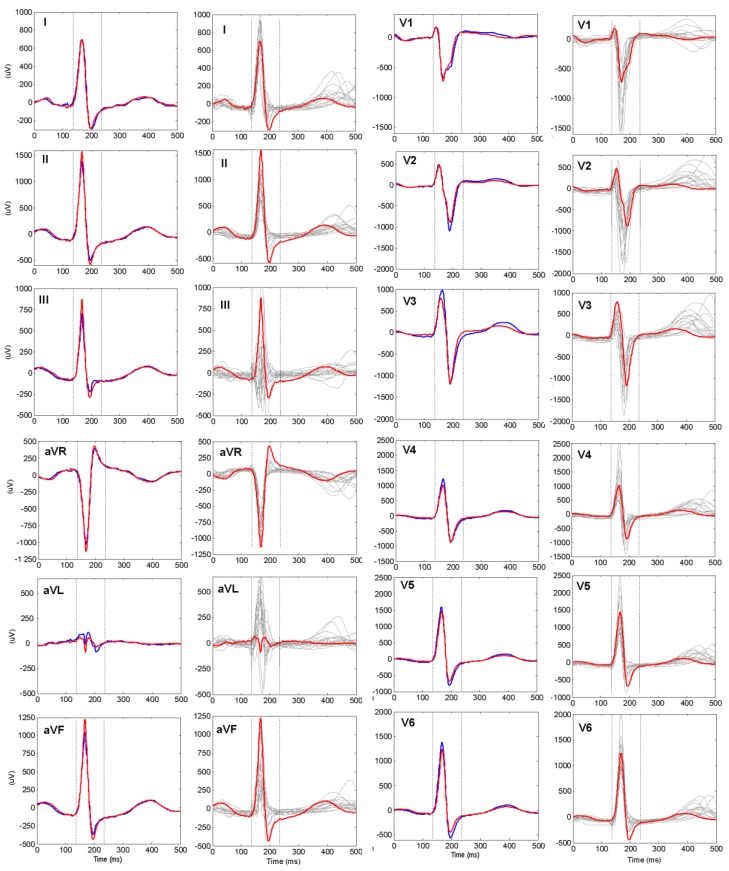
Example of personalized 12-lead PQRST patterns analysed during the ID process. The identity of the tested subject ID_T_ (red line) is examined via comparison to a reference subject ID_R_ with the same identity (1st and 3rd columns, blue line) and 15 different identities (2nd and 4th columns, grey traces). The vertical dotted lines enclose the extracted QRS pattern (100 ms) which is used for the calculation of COR-QRS, AMP-QRS and MATCH-QRS features. COR-PQRST features are estimated over the entire 500 ms segment.

An example of a real human identification scenario based on 12-lead ECG is presented in Figure 3. In all leads we observe close matching between the personalized PQRST and QRS patterns of the same individual at two different time points (red vs. blue trace). In contrast, apparent deviances of the waveforms across different subjects are visible (red vs. grey traces) regardless of the overlapping of certain waveforms. Further in section Results we have presented the training and validation of ID models which deal with inter-subject waveform differences and long-term intra-subject waveform stability in 12-lead ECGs of a large population. 

### 3.1. Statistical Study

The distributions of all features are estimated on the training dataset for 230 equal (ID_T_ = ID_R_) and 230 × 229 = 52670 different (ID_T_ ≠ ID_R_) identity pairs (Table 1). They prove the biometric potential of all features with significantly higher mean values for the groups of equal vs. different subjects (82–96% vs. 60–86%, *p* < 0.0001). This is a precondition to expect distinguishable similarity index ID_ix_ involving all or a selected set of features used in the next phase of training of optimal ID models.

### 3.2. Training of ID Models on 230 Subjects (ID = 1 to 230)

In order to assess the intrinsic identification capability of each feature, the simplest ID models involve one feature in one lead (*FEAT*{*Fi*, *Li*}, where *Fi* = {1}, {2}, ..., {7}; *Li* = {1}, {2}, ..., {12}). The results in Table 2 indicate poor *AccID* if only one feature is included in the ID model (not exceeding 41%). The best identification capability is observed for COR-PQRST and COR-QRS single features in limb leads, while the worst performance (<1%) is observed for AMP-QRS features in all leads.

More complex ID models involve all features from the same feature extraction method (*FEAT*{*Fi*,*Li*}, where *Fi* = {1, 2}, {3, 4}, {5}, {6, 7}; *Li* = {1, 2, …, 12}), to compare the identification capability of COR-PQRST, COR-QRS, AMP-QRS, MATCH-QRS. Table 3 indicates the best identification capability for MATCH-QRS with 15 features (83%), followed by COR-PQRST with 18 features (77%), COR-QRS with 9 features (74%) and the worst AMP-QRS with 7 features (24%). The non-redundant feature sets in the ID models include features that are equally selected from limb and chest leads. 

The next type of ID models involves all features in a single lead set (1 lead, 6 limb leads, 6 chest leads, 12 leads, i.e. *FEAT*{*Fi*,*Li*}, where *Fi* = {1, 2, …, 7}; *Li* = {1}, {2}, ..., {12}, {1, 2, ... 6}, {7 , 8, ... 12}, {1, 2, … 12}). The purpose is to assess the intrinsic identification capability of that specific lead set (Table 4). The identification capability of single limb leads is the lowest in III, aVR (about 47% with up to 5 features) and the highest in I, II (about 57–58% with 5 features). It is increased by 20% when 6 limb leads are used in the ID model (about 77% with 19 features). The identification capability of single chest leads is the lowest in V3 (about 27% with 3 features) and the highest in V1 (about 44% with 4 features). It is increased by 20% when 6 chest leads are used in the ID model (about 64% with 18 features). The most complex ID model with 12-leads and 27 features improves *AccID* to 91.3%, which is about 14% and 27% points better than 6 limb and 6 chest leads, respectively. 

### 3.3. Validation of ID Models on 230 Subjects (ID = 231 to 460)

The identification accuracy strongly depends on the number of reference subjects, i.e. the more the subjects there are, the higher the random match probability is, i.e. to find a subject with better similarity than his own. Therefore, *AccID* worsening is expected for reference database with larger size. We observe this trend for the independent validation set in order to find the number of reference subjects that could be managed by the trained ID models within different *AccID* tolerance ranges. For this purpose, we consider different number of reference subjects and test the ID models with all possible combinations of 10, 20, 30, …, 210, 220, 230 subjects within the total validation set of 230 subjects.

**Figure 4:**
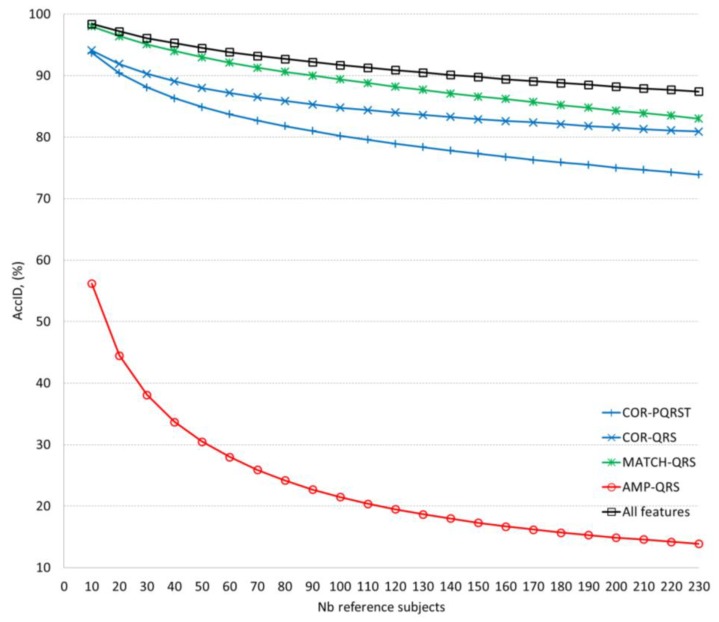
Mean value of the validation *AccID* in function of the number of reference subjects (data in Table 5). The graphs are presented in respect of the feature extraction methods.

Table 5 and Figure 4 compare the validation performance of the optimal ID models for different feature extraction methods using 12-lead ECG. Accepting a confident *AccID* threshold (>90%), we found that it is satisfied for 140, 90, 30, 20 reference subjects by the ID models based on all features, MATCH-QRS, COR-QRS, COR-PQRST, respectively. The AMP-QRS model is limited to maximum 56% achieved just for 10 reference subjects.

**Figure 5:**
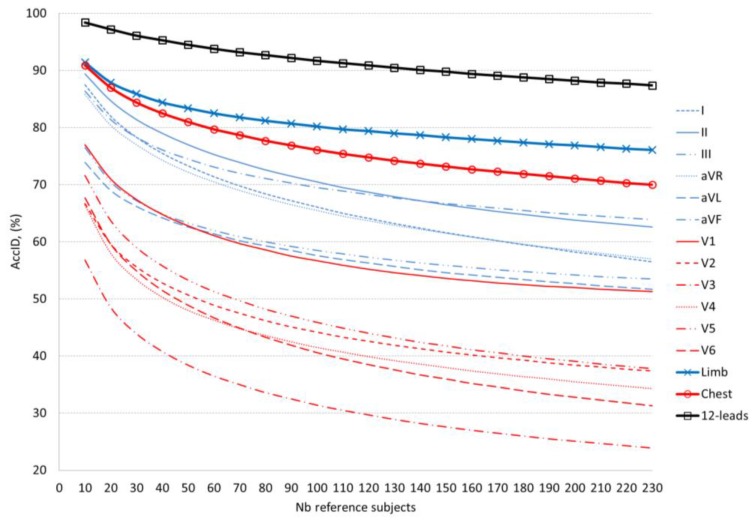
Mean value of the validation *AccID* in function of the number of reference subjects (data in Table 6). The graphs are presented in respect of the ECG lead set implemented in the model: Blue lines: Single limb leads (dashed lines) and all limb leads (solid line); Red lines: Single chest leads (dashed lines) and all chest leads (solid line); Black lines: 12-leads.

Table 6 and Figure 5 compare the validation performance of the optimal ID models for different lead sets. The accepted *AccID* threshold (>90%) is not satisfied for any single lead, even for just 10 reference subjects. The most prominent single limb and chest leads fulfil reduced requirements for *AccID* thresholds. The top-ranked limb lead II achieves *AccID* > 80% for 30 reference subjects and *AccID* > 70% for 100 reference subjects. The top-ranked chest lead V1 achieves only the minor threshold *AccID* > 70% for 20 reference subjects. The multi-lead sets are more powerful and they satisfy higher *AccID* requirements. Identification accuracy *AccID* > 90% is achieved for 10 reference subjects using either 6 chest leads or 6 limb leads and 140 reference subjects when using 12-lead ECG. A lower threshold of *AccID* > 80% is satisfied for 50 reference subjects using 6 chest leads, 100 reference subjects using 6 limb leads and more than 230 reference subjects (maximal population in this study) using 12-lead ECG.

## 4. Discussion

Although being a part of one general biometric task, the aims of human “identification” and human “verification” are quite different, i.e. to recognize an individual in a database by comparing a single input sample to many stored templates and to verify/reject person’s identity by comparing a single input sample to a single stored template. The same features for comparison between input-to-template waveforms could be used in both sub-tasks, however, the decision-making process is different. Usually, the decision for “identification” is performed via the best similarity assessment approach, while the “verification” relies on threshold based techniques. In our recent study [29] we solve the “verification” problem via 6 cross-correlation and 2 amplitude features of QRS and PQRST patterns, involved in a linear discriminant threshold decision function. In this study, we use the same large population to solve the “identification” task adopting 4 cross-correlation and 1 amplitude features and adding 2 more QRS pattern features that measure the similarity in the time and amplitude scales. They are calculated with a brand-new method for binary template matching of short-duration QRS patterns (100 ms). The presented identification model applies a normalized scale for representation of all 7 features (0–100%), where 0% corresponds to the least matching and 100% to the best matching between input and template waveforms. The optimization of the feature selection scheme involved in the calculation of the common similarity index is presented by iterative maximization of the identification accuracy *AccID*.

The questions of greatest concern for the human identification task are: “*Is there an individual whose personalized beat pattern would be more similar to another than to his own in a long-term basis?*”, “*What is the probability to find such an individual within an increasing population of reference subjects?*” 

A quantitative answer to these questions is presented in this study following a straightforward list of training and validation tests which aim to reveal specific aspects of the current human ID investigation. 

(1) *Is there a statistical justification for using the defined features for waveform similarity?*

Table 1 presents the statistics of the features’ distributions in the training dataset, proving that all of them exhibit significantly higher similarity of the waveforms measured across the same individuals in a long-term basis (>1 year) than what can be seen between different individuals (mean value in the range about 82–96% vs. 60–86%, *p* < 0.0001). This proves that the similarity index ID_ix_ defined for finding the best match between subjects could include all features on the same basis. 

(2) *Which are the most reliable QRS and PQRST features for human ID?*

This aspect is investigated by training optimal ID models with different configurations of features using a large dataset with 230 individuals. The simplest model (one-feature in one-lead, Table 2) distinguishes *r*(*lag*0) in leads (aVL, aVF, III) with maximal *AccID* in the range of 35 to 41%. It is about 10% points better than the other distinguished feature *r*(max) where *AccID* ranges between 24 and 35%. The advance of both correlation coefficients is due to different spatial dynamics of the cardiac vector between different subjects. This dissimilarity is enhanced in the zero-lag correlation which additionally represents the temporal desynchronization of the depolarization-repolarization process in different individuals. We suggest that the latter effect is compensated in aVR by using this lead for synchronization of each subject’s average beat to the reference pattern and this explains the remarkably low *AccID* in aVR (11–15%) in respect to the other limb leads. The joint analysis of *r*(*lag*0) and *r*(max) in 12-lead ECG increases *AccID* by about 40% reaching 73.5% (COR-QRS) and 77.4% (COR-PQRST)—see Table 3. These results indicate that the QRS pattern (100 ms) carry the essential subject-specific information, while P and T waves in the PQRST pattern (500 ms) contribute only for a slight ID accuracy improvement by about 4 percentage points. 

The analysis of AMP-QRS shows definitively that the QRS peak-to-peak range across individuals is an indistinguishable feature. It provides *AccID* < 1% for all single leads (Table 2), which increases up to 24% for the optimal ID model (Table 3) including the 7 ECG leads that are the most distinguishable in amplitude (I, II, III, V1, V2, V3, V5). 

The MATCH-QRS method is not powerful when its features are analysed in single leads (maximal accuracy of 26.5% for *EQUA* in aVF, Table 2). This is suggested by the methodological approach for QRS waveform approximation in *binQRS* matrix [100 × 80], which gives indistinguishably high waveform similarity for many subjects whose QRS waveforms are matching within the pre-set time and amplitude tolerances of ±1 ms and ±5%, respectively. The accumulated similarity over 12 leads, however stays high for the same identity subjects and decreases for different identity subjects. Thus, 15 MATCH-QRS measures are selected in the best ID model (*AccID* = 83.5%, Table 3), which represents the best similarity for 10 leads in the amplitude scale (I, II, III, avR, avF, V1, V2, V4, V5, V6) and for 5 leads in the time scale (I, aVR, aVL, V1, V3). 

(3) *Which are the most reliable leads for human ID?*

This aspect is investigated by training optimal ID models with different configurations of leads on a large dataset with 230 individuals (Table 4). In a single lead ID scenario, we highlight limb leads I and II (maximal *AccID* of 58%) followed by aVF (55%) and aVL (50%) including 3 to 5 features from all feature extraction methods. Compared to the best limb leads (I and II), the single chest leads are from 14 to 30% less powerful for the purposes of human ID. They are ranked in the following order: V1 (maximal *AccID* of 44%) followed by V6, V5, V2 (37–35%) and V3, V4 (27–30%). Such a drop in accuracy, especially in anterior leads, could be explained by V1–V6 proximity to the heart, so that small electrode misplacements across different recording sessions of the same individual result in considerable ECG morphology changes seen in chest leads. 

Multi-lead ID models gain considerable raise in accuracy (about 20%) comparing 6 limb leads to the best II (77% vs. 58%) and 6 chest leads to the best V1 (64% vs. 44%). The optimal ID models are built with a prevalent selection of features from leads I, II (6 features), V1, V6 (5 features), aVR, V5 (4 features). The complete ID model, selecting features from all 12-leads, reaches maximal *AccID* of 91.3%. This is better than 6 limb, 6 chest leads and the best single lead (II) with about 14%, 27% and 33% points, respectively. 

(4) *What is the tolerated reference database size for searching human ID?*

This question arises from our previous experience on human identification [23] showing that the time elapsed between the collection of the reference and the test dataset as well as the number of enrolled subjects in the reference dataset have a severe impact on the ID performance. There, *AccID* has been found to drop by 15% (from 93% to 78%) while the population increases by 35 subjects (from 14 to 49). The last is in agreement with [28], reporting *AccID* drop by 10% (from 99% to 89%) for population increase by 40 subjects (from 10 to 50). Naturally, *AccID* worsening could be expected for reference database with larger size, since the more the subjects there are, the higher the random match probability is, i.e. to find a subject with better similarity than his own. According to a comparative study [1], the majority of the published investigations on human ID have been conducted on small populations (about a few dozens of subjects). Considering the missing research information about the influence of the reference database size on the ID accuracy, we present consistent validation of our optimal ID models on an independent dataset by increasing its size from 10 to 230 subjects. Our validation results definitively confirm the expected trend for *AccID* drop with the increase of the number of the reference subjects which however is non-linear and depends on the particular ID model (Figure 4 and Figure 5). We consider as good models those with higher *AccID* and lower *AccID* drop in respect of the number of reference subjects. 

The validation of the ID models applying different feature extraction methods (Table 5, Figure 4) closely reproduces the *AccID* ranges on the training dataset with 230 reference subjects (Table 3). The observed *AccID* differences (training vs. validation) are: COR-PQRST (77% vs. 74%), COR-QRS (74% vs. 81%), AMP-QRS (24% vs. 14%), MATCH-QRS (84% vs. 83%), all features (91% vs. 87%). The differences are relatively small which is a sign of confident training of the ID models with 12-lead ECG capable of adequately evaluating independent database without bias. We observed that the minimal set of 2 MATCH-QRS features is capable of providing human ID with almost the same accuracy (*AccID* > 90%) as the maximal set of all 7 features. However, this is valid for limited reference datasets of up to 100 subjects. More subjects are best identified by the complete ID model including all features which maintains relatively stable performance with a drop of 11% (from 98% to 87%) for population increase by 220 subjects (from 10 to 230). These results outperform the cited in [23,28] which report the same level of *AccID* drop (10–15%) for about 6 times smaller population increase (35–40 subjects). The best performance of our complete ID model could be explained by the fact that larger datasets increase the probability to find similar characteristics across different subjects and thus, more and more robust features are required to distinguish their individuality. 

In a single lead ID scenario (Figure 5, Table 6) we can distinguish two best ID models based on analysis of lead II and aVF for small (10–140) and large (140–230) number of reference subjects, respectively. Overall, even for the best limb leads (II, aVF), we observe a limited ID capability, not exceeding 70% for more than 100 reference subjects and 80% for more than 30 reference subjects. Single chest leads are not usable for human ID. The majority of them (V2–V6) do not exceed 50% for more than 50 subjects. Only V1 can be distinguished with *AccID* above 70% for less than 20 subjects. We recommend multi-lead ID scenarios for management of human search in large populations. Both ID models based on 6 chest and 6 limb leads pass the *AccID* > 70% threshold for the maximal reference dataset (230 subjects) and *AccID* > 80% for up to 100 subjects (limb leads) and up to 50 subjects (chest leads). As expected, 12-lead ECG provides the most robust beat patterns for human ID with *AccID* > 90% for up to 140 subjects, reaching 98.4% for 10 subjects. 

**Figure 6:**
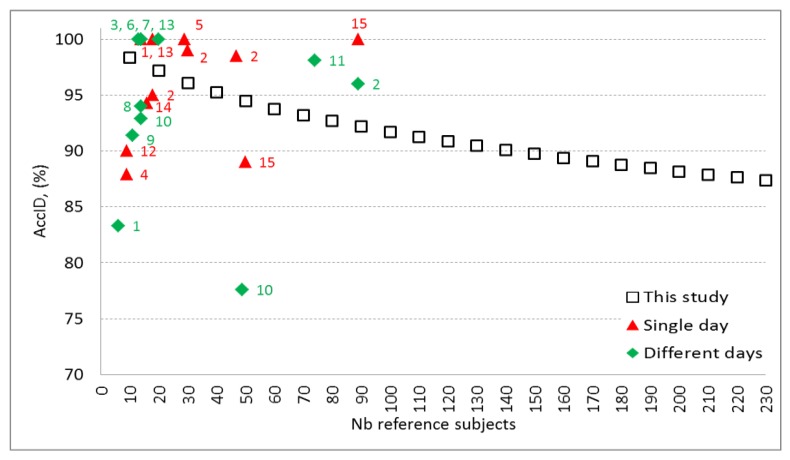
Illustration of the comparative results presented in Table 7. The published studies (from No = 1 to No = 15 in Table 7) are presented in two groups, according to the time distance between the recording sessions, representative for ECG changes in a short (single day) or long-term (different days) basis. For the purpose of adequate comparison, the identification accuracy of our method is illustrated for different number of reference subjects (from 10 to 230 subjects included in the validation set).

The performance of our best ID model (12-leads, 27 features, trained in Table 4, validated in Table 5 and Table 6; Figure 4 and Figure 5) is compared to other published studies on human identification (Table 7, Figure 6).

All published studies do not report training and validation of their ID models on independent datasets (as used in this study), therefore biased *AccID* is probable due to overtraining. For example, such an effect is suspected for studies with accuracy close to 100%, especially on small sized databases [9,16,18,19,20,26]. Overall, the ID studies with enrolment and test phase in a single day report higher *AccID* [9,10,18,26,28]. However, they could not consider longitudinal ECG morphology changes due to physiological or technical sources (e.g. electrode displacement across different sessions). There are two ID studies using multiple sessions in different days on large populations with 74 [24] and 89 subjects [10] that report less than 5 percentage points better performance than our model. This might be due either to superior model algorithm or to the lack of independent validation, considering that the differences between our training and validation accuracies are in the same range. Figure 6 illustrates validation results of this study which extends the knowledge on the ID model performance over the largest span from 10 to 230 reference subjects. The observed decrease of *AccID* could be explained with the appearance of new subjects in the reference dataset who present PQRST waveforms similar to already existing ones. Thus, some of the subjects that match exactly their own pattern in the reference dataset of e.g. 100 subjects show higher correlation and pattern matching with different subject in the reference dataset that contains 230 individuals. This could be due to time-related ECG changes and possible small differences in the electrode placement during the acquisition of the test dataset. Possible approach to keep high *AccID* for larger reference datasets is to include additional robust features in the identification model. However, this should be done very carefully and the designed models should be validated on independent dataset in order to avoid overtraining. Another option to prevent from the assignment of wrong identity to the tested subject is to keep the size of the reference dataset as small as possible for the particular application and to update it with actual PQRST patterns on a regular basis.

## 5. Conclusions

This paper presents a methodology for evaluation of the biometric potential of PQRST pattern waveforms in 12-lead ECG. The focus is on reliable human identification in a large-population database containing two ECGs per patient recorded between 1 to 2 years apart. The benefits from the use of such database are its representativeness for physiologically related long-term ECG changes and effects of possible multi-session technical differences. The investigations demonstrate the identification ability of cross-correlation, amplitude and pattern matching, applied on a personalized heartbeat pattern in single and multi-lead ECG scenarios. The detailed analysis of the identification accuracy highlights the ID model based on cross-correlation and pattern matching of 12-lead ECG as the best one, providing 91.3% identification accuracy over a large training dataset of 230 subjects. The independent validation of this ID model on datasets with different sizes (10–230 subjects) confirms its reliability for human identification with accuracy between 98.4% and 87.4% and extends the knowledge on the ID model performance over a large span of reference subjects.

## 6. Future Work

Up to this moment, our experience in human verification/identification involves the application of fiducial-based and fiducial independent approaches with databases collected with clinical ECG devices. Our future work would revolve around the design of hybrid methods for human verification/identification and their implementation in an identification ECG device prototype, working with a pre-selected minimal lead set.

## Figures and Tables

**Table 1:** Statistical distributions (Mean ± std) of the features’ values averaged for 12-lead ECG in the groups of equal (ID_T_ = ID_R_) and different (ID_T_ ≠ ID_R_) individuals. Statistically significant differences are observed for all features.

Feature Extraction Method	Feature	ID_T_ = ID_R_ (%)	ID_T_ ≠ ID_R_ (%)	*p*-Value
COR-PQRST	*r*(max)	93.81 ± 2.92	81.10 ± 5.93	<0.0001
	*r*(*lag*0)	92.2 ± 3.69	70.01 ± 10.74	<0.0001
COR-QRS	*r*(max)	96.14 ± 2.28	85.99 ± 6.05	<0.0001
	*r*(*lag*0)	93.77 ± 3.90	72.0 ± 12.59	<0.0001
AMP-QRS	*ratioQRS*	83.44 ± 4.87	70.25 ± 7.62	<0.0001
MATCH-QRS	*EQUT*	82.26 ± 5.54	59.54 ± 7.73	<0.0001
	*EQUA*	92.74 ± 2.49	80.59 ± 4.22	<0.0001

**Table 2:** Training *AccID* (%) of each feature in each lead. The colour map distinguishes five *AccID* levels for visual ranking of the feature’s ability for human identification: the worst ≤5%, is given in in red; [5–15)%, in orange; [15–25)%, in yellow; [25–35)%, in light green; and the highest, >35%, in dark green.

Lead	COR-PQRST		COR-QRS		AMP-QRS	MATCH-QRS	
	*r*(max)	*r*(*lag*0)	*r*(max)	*r*(*lag*0)	*ratioQRS*	*EQUT*	*EQUA*
I	20	25.7	15.2	23.0	<1	10.0	23.9
II	23.5	29.6	18.7	24.3	<1	11.3	24.3
III	27.0	37.4	25.2	34.8	<1	5.7	22.2
aVR	15.2	13.0	10.9	12.2	<1	4.3	10.0
aVL	34.8	40.9	31.7	34.8	<1	4.8	24.8
aVF	33.0	39.1	23.5	38.3	<1	8.7	26.5
V1	15.7	21.3	13.0	13.9	<1	1.3	16.1
V2	16.5	20.4	11.7	19.6	<1	6.5	17.8
V3	12.6	19.6	7.0	11.7	<1	1.7	13.9
V4	14.3	20	7.4	12.2	<1	2.6	12.6
V5	11.7	15.7	7.4	8.3	<1	1.7	10.0
V6	13.0	13.5	5.2	7.4	<1	<1	6.5

**Table 3:** Optimal ID models for different feature extraction methods using 12-lead ECG. The non-redundant features and leads included in each model are marked with ‘+’. The training *AccID* (mean ± CI) of each model is reported in the last row. The applied colour map highlights the features involved in a model and the achieved *AccID* as follows: light green for COR-PQRST model; yellow for COR-QRS model; orange for AMP-QRS model and dark green for MATCH-QRS model.

Lead	COR-PQRST		COR-QRS		AMP-QRS	MATCH-QRS	
	*r*(max)	*r*(lag0)	*r*(max)	*r*(lag0)	*ratioQRS*	*EQUT*	*EQUA*
I	+	+	+	+	+	+	+
II	+	+		+	+		+
III	+				+		+
aVR	+			+		+	+
aVL	+	+				+	
aVF	+	+		+			+
V1	+	+	+	+	+	+	+
V2	+	+			+		+
V3	+				+	+	
V4		+					+
V5		+	+		+		+
V6	+		+				+
*AccID* (%)	77.4 ± 5.5		73.5 ± 5.8		23.9 ± 5.5	83.5 ± 4.9	

**Table 4:** Optimal ID models for single- and multi-lead sets. The non-redundant features included in the models are marked: ‘+’ for single leads, ‘*’ for limb leads, ‘o’ for chest leads, ‘#’ for 12-lead ECG. Additionally, the last 3 rows belonging to the multi-lead models show the number of leads per feature, involved in the model. The training *AccID* (mean ± CI) of each model is reported in the last column. The following colour map is applied: the features involved in the single lead models and the achieved *AccID* are highlighted in yellow; the features involved in the model using the 6 chest leads and the respective *AccID* are highlighted in orange; the features involved in the model using the 6 limb leads and its *AccID* are highlighted in light green; and the features involved in the 12-leads model and the achieved *AccID* are highlighted in dark green.

Lead Set	COR-PQRST						COR-QRS						AMP-QRS			MATCH-QRS						
	*r*(max)			*r*(*lag*0)			*r*(max)			*r*(*lag*0)			*ratioQRS*			*EQUT*			*EQUA*			*AccID* (%)
I		*	#	+	*	#	+	*	#	+	*	#	+				*	#	+	*	#	57.8 ± 6.4
II	+	*	#	+	*	#	+	*	#	+	*	#					*	#	+	*		57.4 ± 6.4
III	+			+		#	+			+									+			46.5 ± 6.5
aVR	+	*	#		*		+	*		+	*								+			47.0 ± 6.6
aVL				+	*	#	+				*		+									50.4 ± 6.5
aVF	+			+	*		+		#	+									+			55.2 ± 6.5
V1	+	o	#		o	#	+	o		+	o		+					#		o	#	44.3 ± 6.4
V2			#	+			+	o		+			+							o		34.8 ± 6.2
V3				+			+						+								#	27.4 ± 5.8
V4		o		+			+			+			+							o	#	30.0 ± 6.0
V5		o	#	+	o		+	o	#	+	o		+								#	34.8 ± 6.2
V6	+	o	#	+	o		+	o		+	o									o	#	37.4 ± 6.3
6 Limb	3			5			3			4						2			2			77.4 ± 5.5
6 Chest	4			3			4			3									4			63.9 ± 6.2
12-leads	7			5			4			2						3			6			91.3 ± 3.7

**Table 5:** Validation *AccID* (mean ± CI) of the optimal ID models for different feature extraction methods using 12-lead ECG (Table 3). *AccID* is evaluated for different number of reference subjects (10 to 230 subjects included in the validation set). The colour map is provided for visual identification of the ID models’ ability to keep the mean *AccID* within different tolerance ranges: ≥90% in dark green, [80–90)% in light green, [70–80)% in yellow, [50–70)% in orange, <50% in grey.

Nb Subjects	COR-PQRST	COR-QRS	AMP-QRS	MATCH-QRS	All Features
10	93.7 ± 2.1	94.1 ± 2.4	56.2 ± 4.7	98.0 ± 0.8	98.4 ± 0.8
20	90.4 ± 2.9	91.9 ± 2.9	44.5 ± 5.0	96.4 ± 1.4	97.2 ± 1.3
30	88.1 ± 3.3	90.3 ± 3.2	38.1 ± 4.9	95.1 ± 1.9	96.1 ± 1.7
40	86.3 ± 3.6	89.1 ± 3.4	33.7 ± 4.8	94.0 ± 2.2	95.3 ± 2.0
50	84.9 ± 3.9	88.0 ± 3.6	30.5 ± 4.8	93.0 ± 2.5	94.5 ± 2.2
60	83.7 ± 4.1	87.2 ± 3.8	28.0 ± 4.7	92.1 ± 2.7	93.8 ± 2.4
70	82.7 ± 4.2	86.5 ± 3.9	25.9 ± 4.7	91.3 ± 2.8	93.2 ± 2.6
80	81.8 ± 4.4	85.9 ± 4.1	24.2 ± 4.6	90.6 ± 3.0	92.7 ± 2.7
90	81.0 ± 4.5	85.3 ± 4.2	22.7 ± 4.6	90.0 ± 3.2	92.2 ± 2.9
100	80.2 ± 4.6	84.8 ± 4.3	21.5 ± 4.5	89.4 ± 3.3	91.7 ± 3.0
110	79.6 ± 4.7	84.4 ± 4.3	20.4 ± 4.5	88.8 ± 3.4	91.3 ± 3.1
120	78.9 ± 4.8	84.0 ± 4.4	19.5 ± 4.5	88.2 ± 3.6	90.9 ± 3.3
130	78.4 ± 4.9	83.6 ± 4.5	18.7 ± 4.5	87.7 ± 3.7	90.5 ± 3.4
140	77.8 ± 5.0	83.3 ± 4.6	18.0 ± 4.5	87.1 ± 3.8	90.1 ± 3.5
150	77.3 ± 5.1	82.9 ± 4.6	17.3 ± 4.5	86.6 ± 3.9	89.8 ± 3.6
160	76.8 ± 5.1	82.6 ± 4.7	16.7 ± 4.5	86.2 ± 4.0	89.4 ± 3.7
170	76.3 ± 5.2	82.4 ± 4.7	16.2 ± 4.4	85.7 ± 4.2	89.1 ± 3.8
180	75.9 ± 5.3	82.1 ± 4.8	15.7 ± 4.4	85.2 ± 4.3	88.8 ± 3.9
190	75.5 ± 5.4	81.8 ± 4.9	15.3 ± 4.4	84.8 ± 4.4	88.5 ± 3.9
200	75.0 ± 5.5	81.6 ± 4.9	14.9 ± 4.5	84.3 ± 4.5	88.2 ± 4.0
210	74.7 ± 5.5	81.3 ± 5.0	14.6 ± 4.5	83.9 ± 4.6	87.9 ± 4.1
220	74.3 ± 5.6	81.1 ± 5.0	14.2 ± 4.5	83.5 ± 4.7	87.7 ± 4.2
230	73.9 ± 5.7	80.9 ± 5.1	13.9 ± 4.5	83.0 ± 4.9	87.4 ± 4.3

**Table 6:** Validation *AccID* (mean ± CI, %) of the optimal ID models for single- and multi-lead sets (Table 4). *AccID* is evaluated for different number of reference subjects (10 to 230 subjects included in the validation set). The colour map is provided for visual identification of the ID models’ ability to keep the mean *AccID* within different tolerance ranges: ≥90% in dark green, [80–90)% in light green, [70–80)% in yellow, [50–70)% in orange, <50% in grey. The bolded values correspond to the top ranked *AccID* across limb leads (II for 1–140 subjects, aVF for 140–230 subjects), chest leads (V1) and multi-lead sets (12-leads).

Nb Subjects	I	II	III	aVR	aVL	aVF	V1	V2	V3	V4	V5	V6	Limb	Chest	12-Leads
10	87.5 ± 3.1	**89.4 ± 2.9**	76.5 ± 4.5	85.8 ± 3.4	73.9 ± 4.9	86.4 ± 3.4	**77.0 ± 4.5**	66.7 ± 5.1	56.8 ± 5.3	66.4 ± 4.9	71.6 ± 4.6	67.7 ± 4.9	91.4 ± 2.8	90.9 ± 2.7	**98.4 ± 0.8**
20	82.0 ± 3.8	**84.7 ± 3.6**	70.5 ± 5.1	80.4 ± 4.1	69.0 ± 5.3	81.4 ± 4.1	**71.0 ± 5.0**	59.6 ± 5.5	48.5 ± 5.5	58.0 ± 5.4	63.7 ± 5.1	59.6 ± 5.3	87.9 ± 3.5	87.0 ± 3.5	**97.2 ± 1.3**
30	78.3 ± 4.3	**81.4 ± 4.0**	67.2 ± 5.4	77.0 ± 4.5	66.2 ± 5.5	78.3 ± 4.5	**67.4 ± 5.2**	55.6 ± 5.6	43.9 ± 5.5	53.4 ± 5.6	59.0 ± 5.4	54.9 ± 5.4	85.9 ± 3.9	84.4 ± 3.9	**96.1 ± 1.7**
40	75.5 ± 4.6	**79.0 ± 4.3**	64.9 ± 5.6	74.3 ± 4.7	64.2 ± 5.6	76.1 ± 4.8	**64.8 ± 5.4**	52.8 ± 5.7	40.8 ± 5.5	50.3 ± 5.7	55.8 ± 5.5	51.5 ± 5.5	84.4 ± 4.2	82.5 ± 4.2	**95.3 ± 2.0**
50	73.3 ± 4.8	**77.0 ± 4.5**	63.3 ± 5.7	72.2 ± 4.9	62.6 ± 5.7	74.5 ± 5.0	**62.8 ± 5.5**	50.7 ± 5.7	38.4 ± 5.5	48.0 ± 5.7	53.3 ± 5.6	48.9 ± 5.5	83.4 ± 4.3	81.0 ± 4.4	**94.5 ± 2.2**
60	71.4 ± 4.9	**75.3 ± 4.7**	62.0 ± 5.8	70.5 ± 5.1	61.4 ± 5.7	73.1 ± 5.1	**61.1 ± 5.6**	48.9 ± 5.8	36.5 ± 5.4	46.3 ± 5.8	51.3 ± 5.7	46.7 ± 5.5	82.5 ± 4.5	79.7 ± 4.5	**93.8 ± 2.4**
70	69.8 ± 5.1	**73.9 ± 4.9**	60.9 ± 5.8	69.0 ± 5.2	60.2 ± 5.8	72.0 ± 5.3	**59.7 ± 5.7**	47.5 ± 5.8	35.0 ± 5.4	44.8 ± 5.8	49.7 ± 5.7	44.9 ± 5.6	81.8 ± 4.6	78.7 ± 4.7	**93.2 ± 2.6**
80	68.4 ± 5.2	**72.6 ± 5.0**	60.0 ± 5.9	67.7 ± 5.3	59.3 ± 5.9	71.1 ± 5.4	**58.6 ± 5.8**	46.2 ± 5.8	33.6 ± 5.4	43.6 ± 5.8	48.2 ± 5.8	43.3 ± 5.6	81.2 ± 4.7	77.7 ± 4.8	**92.7 ± 2.7**
90	67.2 ± 5.3	**71.5 ± 5.1**	59.2 ± 5.9	66.5 ± 5.4	58.5 ± 5.9	70.3 ± 5.4	**57.5 ± 5.9**	45.1 ± 5.9	32.5 ± 5.4	42.5 ± 5.8	47 ± 5.8	41.9 ± 5.6	80.7 ± 4.7	76.9 ± 4.9	**92.2 ± 2.9**
100	66.1 ± 5.4	**70.5 ± 5.3**	58.5 ± 6.0	65.5 ± 5.5	57.6 ± 6.0	69.5 ± 5.5	**56.7 ± 6.0**	44.2 ± 5.9	31.4 ± 5.4	41.5 ± 5.8	45.9 ± 5.8	40.6 ± 5.6	80.2 ± 4.8	76.1 ± 5.0	**91.7 ± 3.0**
110	65.0 ± 5.5	**69.5 ± 5.4**	57.9 ± 6.0	64.5 ± 5.6	56.9 ± 6.0	68.9 ± 5.6	**55.9 ± 6.0**	43.3 ± 5.9	30.5 ± 5.4	40.7 ± 5.9	44.9 ± 5.9	39.5 ± 5.7	79.7 ± 4.9	75.4 ± 5.1	**91.3 ± 3.1**
120	64.1 ± 5.6	**68.7 ± 5.5**	57.3 ± 6.1	63.7 ± 5.7	56.3 ± 6.0	68.3 ± 5.6	**55.2 ± 6.1**	42.6 ± 6.0	29.7 ± 5.4	39.9 ± 5.9	44.0 ± 5.9	38.5 ± 5.7	79.4 ± 4.9	74.8 ± 5.2	**90.9 ± 3.3**
130	63.2 ± 5.7	**67.9 ± 5.5**	56.8 ± 6.1	62.9 ± 5.7	55.7 ± 6.1	67.7 ± 5.7	**54.6 ± 6.1**	41.9 ± 6.0	28.9 ± 5.4	39.2 ± 5.9	43.2 ± 5.9	37.6 ± 5.7	79.0 ± 5.0	74.2 ± 5.3	**90.5 ± 3.4**
140	62.4 ± 5.7	**67.2 ± 5.6**	56.3 ± 6.2	62.2 ± 5.8	55.1 ± 6.1	**67.2 ± 5.8**	**54.1 ± 6.2**	41.3 ± 6.0	28.2 ± 5.4	38.6 ± 5.9	42.4 ± 6.0	36.7 ± 5.7	78.7 ± 5.0	73.7 ± 5.3	**90.1 ± 3.5**
150	61.6 ± 5.8	66.5 ± 5.7	55.9 ± 6.2	61.5 ± 5.9	54.6 ± 6.2	**66.7 ± 5.8**	**53.6 ± 6.2**	40.7 ± 6.0	27.6 ± 5.4	38.0 ± 5.9	41.8 ± 6.0	36.0 ± 5.8	78.3 ± 5.1	73.2 ± 5.4	**89.8 ± 3.6**
160	60.9 ± 5.9	65.9 ± 5.8	55.5 ± 6.2	60.8 ± 5.9	54.2 ± 6.2	**66.3 ± 5.9**	**53.2 ± 6.3**	40.2 ± 6.1	27.0 ± 5.4	37.4 ± 6.0	41.1 ± 6.0	35.2 ± 5.8	78.0 ± 5.1	72.7 ± 5.5	**89.4 ± 3.7**
170	60.2 ± 6.0	65.3 ± 5.9	55.1 ± 6.3	60.2 ± 6.0	53.8 ± 6.3	**65.9 ± 5.9**	**52.8 ± 6.3**	39.7 ± 6.1	26.5 ± 5.4	36.9 ± 6.0	40.6 ± 6.1	34.6 ± 5.8	77.7 ± 5.2	72.3 ± 5.5	**89.1 ± 3.8**
180	59.5 ± 6.0	64.8 ± 5.9	54.8 ± 6.3	59.6 ± 6.1	53.4 ± 6.3	**65.5 ± 6.0**	**52.5 ± 6.3**	39.3 ± 6.1	26.0 ± 5.4	36.4 ± 6.0	40.0 ± 6.1	33.9 ± 5.8	77.4 ± 5.3	71.9 ± 5.6	**88.8 ± 3.9**
190	58.9 ± 6.1	64.3 ± 6.0	54.5 ± 6.3	59.0 ± 6.1	53.0 ± 6.3	**65.1 ± 6.0**	**52.2 ± 6.4**	38.8 ± 6.1	25.5 ± 5.4	36.0 ± 6.0	39.5 ± 6.1	33.3 ± 5.9	77.1 ± 5.3	71.5 ± 5.7	**88.5 ± 3.9**
200	58.2 ± 6.2	63.8 ± 6.1	54.2 ± 6.4	58.5 ± 6.2	52.7 ± 6.4	**64.8 ± 6.1**	**52.0 ± 6.4**	38.4 ± 6.2	25.1 ± 5.5	35.5 ± 6.1	39.1 ± 6.2	32.8 ± 5.9	76.9 ± 5.4	71.1 ± 5.7	**88.2 ± 4.0**
210	57.7 ± 6.3	63.4 ± 6.1	53.9 ± 6.4	58.0 ± 6.3	52.3 ± 6.4	**64.5 ± 6.1**	**51.7 ± 6.4**	38.1 ± 6.2	24.7 ± 5.5	35.1 ± 6.1	38.6 ± 6.2	32.3 ± 5.9	76.6 ± 5.4	70.7 ± 5.8	**87.9 ± 4.1**
220	57.1 ± 6.3	63.0 ± 6.2	53.7 ± 6.4	57.5 ± 6.3	52.0 ± 6.4	**64.2 ± 6.2**	**51.5 ± 6.4**	37.7 ± 6.2	24.3 ± 5.5	34.7 ± 6.1	38.2 ± 6.2	31.8 ± 6.0	76.3 ± 5.5	70.3 ± 5.9	**87.7 ± 4.2**
230	56.5 ± 6.4	62.6 ± 6.3	53.5 ± 6.5	57.0 ± 6.4	51.7 ± 6.5	**63.9 ± 6.2**	**51.3 ± 6.5**	37.4 ± 6.3	23.9 ± 5.5	34.3 ± 6.2	37.8 ± 6.3	31.3 ± 6.0	76.1 ± 5.5	70.0 ± 5.9	**87.4 ± 4.3**

**Table 7:** Comparative study of *AccID* for our best ID model (validation results) and the results reported by other authors over databases with different number of subjects (the databases’ abbreviations are cited according to their reference in the source publication). The information about single or multiple recordings per patient (srpp or mrpp) and the interval between T1 and T2 (in case of mrpp) is provided.

No	Study	Database (DB)	Nb Subjects	Recording Sessions	*AccID* (%)
1	[9]	PTB DB: Test set 1	14	single day (mrpp)	100
		PTB DB: Test set 2	6	different days (mrpp, several months)	83.3
2	[10]	Physionet human-ID	89	mrpp, 16 months	96
		MIT-BIH (NSR)	18	srpp	95
		MIT-BIH (Arrhythmia)	47	srpp	98.5
		Own DB (Mobile phone)	30	srpp	99
3	[16]	Own DB	20	different days (mrpp, 6 weeks)	100
4	[17]	Own DB	9	mrpp, single day	87.9 *
5	[18]	Own DB	29	mrpp, single day	100
6	[19]	PTB DB	14	mrpp, from single day to several months	100
7	[20]	PTB DB	14	mrpp, from single day to several months	100
8	[21]	Own DB	14	mrpp, 16 months	94
9	[22]	Own DB	11	mrpp, 16 months	91.4
10	[23]	ILSA DB	49	mrpp, 5 years	77.6
		PTB DB	14	mrpp, from single day to several months	92.9
11	[24]	Own DB	74	mrpp, 16 months	98.1
12	[25]	Own DB	9	srpp	90
13	[26]	MIT-BIH	18	srpp	100
		PTB DB	13	mrpp, from single day to several months	100
14	[27]	Own DB	16	srpp	94.3
15	[28]	MIT-BIH	89	srpp	100
		Own DB	50	srpp	89
16	This study	Own DB	10	mrpp, 1 to 2 years	98.4
			20	mrpp, 1 to 2 years	97.2
			50	mrpp, 1 to 2 years	94.5
			100	mrpp, 1 to 2 years	91.7
			230	mrpp, 1 to 2 years	87.4

* Note: *AccID* is originally reported as the proportion of beats correctly assigned to the respective patient, in the range between 76.1% and 99.6%. The mean value is used for comparison.
